# Characterization of an Egyptian *Spodoptera littoralis *nucleopolyhedrovirus and a possible use of a highly conserved region from polyhedrin gene for nucleopolyhedrovirus detection

**DOI:** 10.1186/1743-422X-5-13

**Published:** 2008-01-23

**Authors:** AlaaEddeen M Seufi

**Affiliations:** 1 Department of Entomology, Faculty of Science, Cairo University, Giza, 12211, Egypt

## Abstract

An Egyptian isolate of *Spodoptera littoralis *nucleopolyhedrovirus (*Spli*NPV) was tested for its potential as biocontrol agent in comparison to *Autographa californica *multiple nucleopolyhedrovirus (*Ac*MNPV). Comparative assays of *Spli*NPV and *Ac*MNPV against 2^nd ^instar larvae of *Spodoptera littoralis *revealed 4-fold greater susceptibility of *S. littoralis *to *Ac*MNPV than to *Spli*NPV based on LC_50 _values for the two viruses. The LT_50_s determined for *Spli*NPV and *Ac*MNPV using LC_50 _of the virus against 2^nd ^instar larvae were 4.2 and 5.8 days, respectively. A DNA segment of 405 bp containing highly conserved region from polyhedrin gene of *Spli*NPV (*Polh-cr*) was successfully amplified by PCR. Subsequently, this DNA segment was cloned and sequenced. Nucleotide sequence and its deduced amino acid sequence were compared to all available sequences in GenBank. Sequence alignment results revealed that *Polh-cr *showed significant similarities with 91 different baculovirus isolates. The percentage of homology ranged from 78% for *Plusia orichalcea *NPV to 99% for *Spli*NPV. This highly conserved region provides a candidate that could be used in easy, fast and economic prospective systems for virus detection as well as in biological control strategies.

## Introduction

Baculoviruses are considered to be the largest and most broadly studied insect viruses. Although they infect over 600 species of insects [1], individual isolates normally show a limited host range and infect only closely related species. It is believed that baculoviruses are potentially useful as safe biological control agent and in some cases they were used successfully to control different insect pests [2-4]. However the overall use of baculoviruses for biological control is limited compared to other pest control means [3,5]. This is due to **the virulence **and **speed of action**, as related to **dose**, **host range**, **cost of production **and **patent registration **are important effectiveness-determining properties of insect-pathogenic biocontrol agents.

Baculoviruses were not only isolated from the insect orders Hymenoptera, Diptera and Trichoptera, but also were isolated from the crustacean order Decapoda (shrimps) [6]. Furthermore, some NPVs (*Penaeus monodon*-NPV) are considered as serious pests for marine crustaceans [7]. The infection of marine crustaceans (shrimps, prawns, crabs, eustacosa...etc.) with NPV will reduce their economic value and thus **negatively affecting the national income**. This problem will arise in countries that depend on marine wealth as a source of national income and/or where marine crustaceans are a major in most of the peoples' food (Australia and Far East nations like Japan, China, etc...). Although, the shrimp viruses have now been classified as *Nimaviridae *in the genus *Whispovirus *and are no longer baculoviruses [8], their diagnosis by PCR using primers of the polyhedrin and DNA polymerase genes of *Autographa californica *nucleopolyhedrovirus (*Ac*MNPV) and *Lymantria dispar *nucleopolyhedrovirus (*Ld*MNPV) was confirmed by Hsu *et al. *(2000). For the above mentioned reasons, many authors were interested in developing an accurate and easy diagnostic method to detect baculoviral infection in larval shrimps as well as insects [9,7-19].

Because baculoviruses are of great interest and utility to a large cross-sections of **agricultural and biomedical research community**, selection of some isolates with natural improved characteristics and definitive characterization of them have appeared recently. Consequently this piece of work will help in **viral epidemiology**, in **monitoring the viral dissemination **after field application and in **risk assessment **studies.

Therefore, the main objective of the present work is to obtain a sequenced highly conserved DNA fragment from our NPV isolate to be used in an easy, fast and economic prospective system for virus detection. Also, this DNA segment can be used for developing kits of ELISA, hybridization, Western and dot blotting. Furthermore, our NPV isolate may be useful as potential biocontrol agent in programs of integrated pest management (IPM).

## Methods

### Insects

Laboratory colony of the cotton leaf worm, *Spodoptera littoralis*, was originally collected from Giza, Egypt and maintained in the insectary of Agricultural Genetic Engineering Research Institute (AGERI) under highly controlled conditions from 1990 to date. The colony was maintained in the laboratory according to the technique described by El-Defrawi and coworkers [20]. Larvae were reared on a semisynthetic diet described by Levinson and Navon [21]. This colony was kept at 25 ± 2°C, 65–70% RH and natural photoperiod. These insects were used for viral propagation and bioassays.

### Viruses

An NPV isolate was selected from thirteen Egyptian baculovirus isolates from different geographical localities during 1996–2000. This isolate was studied in detail to determine its insecticidal activity compared to *Ac*MNPV. In addition, a highly conserved region of polyhedrin gene was amplified, cloned and sequenced for further investigations. The selected isolate was collected from an Egyptian field located at Khorshid, Alexandria. It was isolated during Jan, 1999 from a *S. littoralis *larva on cabbage plants. This isolate was successfully propagated, purified following the method described by Lacey and coworkers [22] and used for further studies.

A virus stock of NPV from *Autographa californica *nuclear polyhedrosis virus (*Ac*MNPV; E_2 _strain) was originally obtained from Prof. Dr. Suzanne Thiem, Department of Entomology, University of Michigan, was used in the bioassays as a reference in comparison to our isolate.

### Insecticidal activity

Laboratory bioassay tests were conducted using highly purified virus suspensions. The *Spli*NPV and *Ac*MNPV isolates were tested against 2^nd ^larval instar from the virus-free rearing culture in the laboratory. The concentrations were measured using Thoma haemocytometer and light microscope [22]. Bioassays were performed using five concentrations of each virus isolate. Tested larvae were starved for 8 hours prior to feeding viruses. Serial dilutions of each tested isolate were prepared beginning with the following stock concentrations in PIB/ml: 7.8 × 10^4 ^and 9.4 × 10^4 ^for *Spli*NPV and *Ac*MNPV, respectively. Bioassays were carried out in plastic cups measuring 15 cm diameter × 10 cm height and containing a layer of 1 cm semi-synthetic diet. The viral suspensions were dispersed on the diet using micropipette (25 μl suspension/larva). Forty larvae were used for each concentration of virus and control. The control larvae were fed on diet treated with distilled water. All treatments were kept at 25 ± 2°C and normal photoperiod. The experiment was replicated thrice for each virus isolate and control. Mortality was recorded on the 7^th ^day post-infection then corrected according to Abbott's formula [23]. Cumulative mortality was recorded daily and the experiment was stopped on day 10 post-infection. Mortality was corrected according to Abbott's formula [23].

### Statistical analyses

Probit analysis of mortality data from bioassays was conducted using SPSS (ver10.0) computer software (SPSS for Windows, SPSS Inc., 1997). The LT values for the tested isolates were derived from analysis of data on the progression of mortality of *S. littoralis *larvae, following exposure to the LC_50_s dosage using probit analysis modified for multiple observations over time [24] and Mathematica software (Wolfram, Champaign, IL).

### PCR amplification

PCR amplification was performed according to Saiki and coworkers [25] with minor modifications. Total DNA was extracted from the NPV isolate and the DNA segment was amplified using two primers designed based on conserved nucleotide sequences of ten different polyhedrin genes [26]. The forward primer: GG(GT) CC(GT) GGC AAG AAT CAG AA and the reverse one: GCG TC(TG) GG(TG) GCG AAC TCT TT(TG) ATT TT. Total reaction volume was 50 μl which contained 1× PCR buffer (Promega), 1.5 mM MgCl_2_, 200 μM dNTPs, 2.5 U *Taq *DNA polymerase (Promega), 100 ng of each primer and 30 ng of template DNA. The amplification program used was 3 min at 94°C (hot start), 1 min at 94°C, 2 min at 55°C and 2 min at 72°C for 35 cycles followed by one cycle of 72°C for 7 min. PCR amplification was carried out in a DNA thermal cycler (Model 380 A, Applied Biosystems, CA, USA).

### Cloning of a highly conserved region from *Spli*NPV polyhedrin gene

The positive PCR products were visualized and eluted from the gel using GenClean Kit (Invitrogen Corporation, San Diego, CA, USA) as described by the manufacturer. The purified PCR products and a *p*GEM-T vector (Promega Corporation, Madison, WI, USA) were mixed in a 5: 1 (insert: vector) molar ratio and ligated using T_4 _DNA ligase (as described by the manufacturer). Ligation mix was used to transform competent *Escherichia coli *JM_109 _cells (Stratagene, La Jolla, CA, USA). White colonies were screened using PCR as described earlier in this section.

### Nucleotide sequence and sequence analysis

Only one clone *pG*NPV-95 was selected and sequenced using M_13 _universal forward and reverse primers. Sequencing was performed using T^7^SequencingT^M ^kit (Pharmacia, Biotech) and model 310 automated sequencer (Applied Biosystems, Foster City, CA, USA). Analysis of nucleotide and deduced amino acid sequences was carried out using EditSeq-DNAstar Inc., Expert Sequence Analysis software, Windows 32 Edit Seq 4.00 (1989–1999) and ExPasy database on the internet. Blast search for alignment of the obtained sequence with the published ones was done using database of National Centre for Biotechnology Information (NCBI). The cloned DNA fragment (*Polh-cr*) was deposited in GenBank under the AY442260 accession number.

## Results

### Field survey

During the present study, the most promising isolate, as a biocontrol agent (based on LC and LT values), was selected from thirteen Egyptian baculovirus isolates. Through four visits to the location, out of 63 collected *S. littoralis *larvae, 18 larvae were diseased (showed symptoms of viral infection). The virus was diagnosed using light microscope, propagated in a *S. littoralis *laboratory colony, purified and kept at -80°C for further studies.

### Insecticidal activity

Table (1) presents the LC and LT values for the two tested isolates (*Spli*NPV and *Ac*MNPV). It is clear that the newly molted 2^nd ^larval instars of *S. littoralis *were susceptible to the applied concentrations. Based on LC_50 _in PIB/ml, our *Spli*NPV isolate was significantly (4-fold) less active (LC_50 _= 1.2 × 10^3^) than the reference strain *Ac*MNPV (LC_50 _= 3.7 × 10^2^). Although LC_25 _and LC_95 _of our isolate were approximately 2 and 8-fold lower than that of *Ac*MNPV, there were substantial overlap in the 95% confidence limits of the two viruses. Time to death showed some dependence on the initial concentration. Furthermore, it was observed that the highest peak of mortality was on 4–6 days post-infection. The progression of mortality in 2^nd ^instar larvae of *S. littoralis *over a 10-day-period following exposure to LC_50 _dosage of the two viruses was analyzed to produce LT values (Table 1). Probit analysis of the LT values revealed that the decrease in LT values in favour of our isolate against *Ac*MNPV was not significant. Despite the overlap in the 95% confidence intervals of the two viruses, LT_95 _of our isolate was nearly 4 days less than that of *Ac*MNPV (95% c.i. 9.7–25.4 days, *X*^2 ^= 2.64, DF = 8, P = 0.96, slope = 3.49 ± 0.74).

**Table 1:** Susceptibility of 2nd instar larvae of *S. littoralis *to the nucleopolyhedroviruses of *A. californica *(*Ac*MNPV) and *S. littoralis *(*Spli*NPV)

**Isolate**	**LC values in PIB/ml (95% confidence limits)**			**LT values in days (95% confidence limits)**		
	
	**LC**_25_	**LC**_50_*	**LC**_95_	**LT**_25_	**LT**_50_	**LT**_95_
***Ac*MNPV**	19.5 (4.8 – 4.0 × 10)	3.7 × 10^2 ^(1.9 × 10^2 ^– 6.7 × 10^2^)	7.5 × 10^5 ^(2.4 × 10^5 ^– 3.7 × 10^6^)	3.7 (2.50 – 4.59)	5.8 (4.73 – 7.14)	17.2 (12.01 – 39.03)
***Spli*NPV**	39.3 (1.4 × 10 – 8.2 × 10)	1.2 × 10^3 ^(6.9 × 10^2 ^– 2.3 × 10^3^)	6.1 × 10^6 ^(4.5 × 10^5 ^– 1.9 × 10^7^)	2.6 (1.65 – 3.39)	4.2 (3.26 – 5.17)	13.4 (9.74 – 25.42)

Significant difference at 95% confidence interval

### PCR amplification

**Figure 1:**
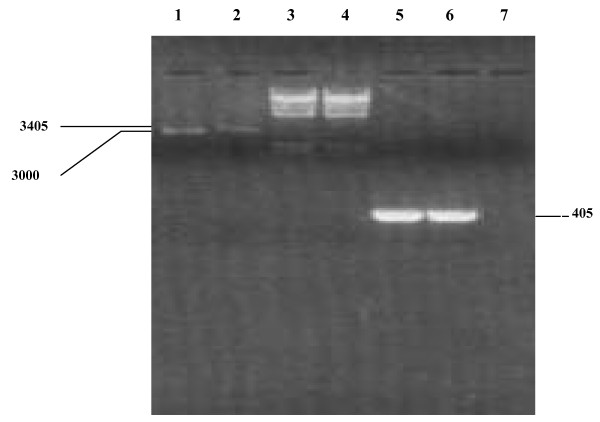
Agarose gel electrophoresis showing clone *p*GNPV-95 after linearization with *Nde*I, and PCR confirmation. Lanes 1 and 2 show *p*GEM-T and *p*GNPV-95 after digestion with *Nde*I, respectively. Lanes 3 and 4 show Lambda DNA/*Hind*III Marker. Lanes 5, 6 and 7 show the 405 bp amplified DNA segment from *Spli*NPV (as positive control), from *E. coli *harbouring *p*GNPV-95 and PCR mix without DNA (as negative control), respectively. The size of the bands is shown in bp.

Two oligonucleotide primers were designed to amplify 537 bp within the open reading frame (*orf*) of polyhedrin gene [26] and were successfully used in PCR. PCR analysis of our results revealed that a DNA fragment of only 405 bp was amplified within the *orf *(beginning about 78 – 81 codons after the starting codon, AUG) of the *Spli*NPV-95 polyhedrin gene (Fig. 1, lane 5). Subsequently this DNA segment was cloned into *p*GEM-T vector (Fig. 1, lane 2) and transformed cells were tested with PCR using the same primers (Fig. 1, lane 6). Using this screening method, clone *p*GNPV-95 was tested as positive (Fig. 1, lane 6).

### Nucleotide sequence and sequence analyses

**Figure 2:**
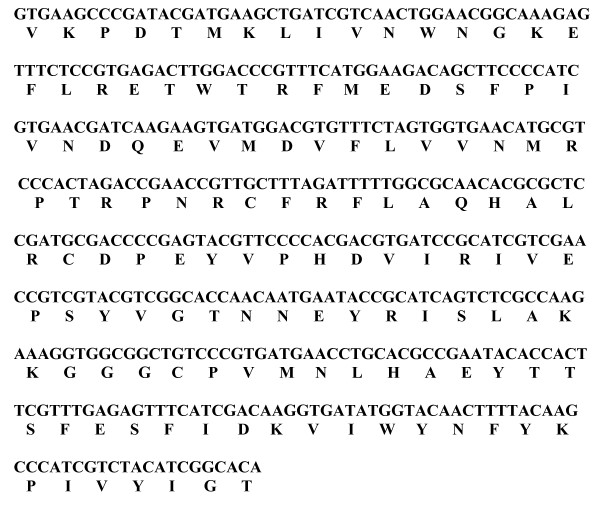
Nucleotide and corresponding deduced amino acid sequence of a highly conserved region of polyhedrin gene (*polh-cr*).

The nucleotide sequence of *Polh-cr *and its deduced amino acid sequence are shown in Fig (2). A single open reading frame (*orf*) that could encode a polypeptide of 135 amino acids was detected. No stop codon was found all over the sequence. This deduced polypeptide contains 16 strongly basic, 16 strongly acidic, 46 hydrophobic and 34 polar amino acids. The calculated molecular mass of the putative polypeptide is 15.92 KDa. Isoelectric point (PI) is 7.242 and the charge at pH 7.0 is 0.331.

The nucleotide sequence of *Polh-cr *was blasted in GenBank database and compared to all available sequences. Alignment results revealed that *Polh-cr *has significant alignment with 111 baculovirus isolates (100 NPVs and 11 GVs). The percentage of homology ranged between 99% for *Spli*NPV (Acc# D01017) and 78% for *Plusia orichalcea *NPV (Acc# AF019882). Interestingly *Polh-cr *produced significant alignments with 11 granulovirus sequences. This may raise the question of possible homologous recombination between NPV and GV species.

**Figure 3:**
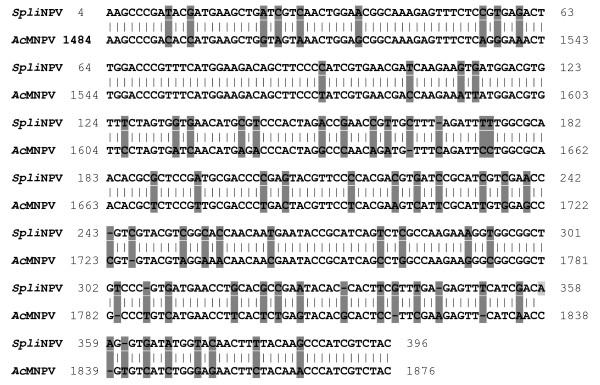
Comparison of *Polh-cr *nucleotide sequence (Acc# AY442260) to its corresponding sequence of *Ac*MNPV (Acc# M25054) as a reference. Gaps and different nucleotides are shaded.

Comparing *Polh-cr *nucleotide sequence (Acc# AY442260) to its corresponding sequence of *Ac*MNPV (Acc# M25054) as a reference, 82% homology, 57 different nucleotides and 12 gaps were observed throughout the compared DNA segments (Fig 3).

The deduced amino acid sequence was compared to all other polyhedrins in GenBank database. Alignment results showed that the percentage of homology of *Polh-cr *ranged between 100% for *S. littoralis *polyhedrins (Acc# AAC33752 and AAR04375) and 81% for *Attacus ricini *polyhedrin (Acc# P31036).

**Figure 4:**
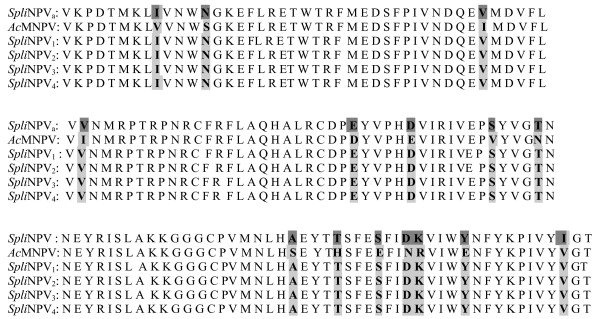
Comparison of amino acid sequence of the putative polypeptide of *Polh-cr *(Acc# AAR04375) to its corresponding sequences of *Ac*MNPV (Acc# AAA46736) and other 4 *Spli*NPVs (Acc# AAW49208, BAA00824, AAT10182 and AAW49207, respectively). The different amino acids were bolded and shaded using *Ac*MNPV as a reference.

On comparing amino acid sequence of the putative polypeptide of *Polh-cr *(Acc# AAR04375) to its corresponding sequences of *Ac*MNPV (Acc# AAA46736) and other 4 *Spli*NPVs (Acc# AAW49208, BAA00824, AAT10182 and AAW49207), 15 different amino acids were observed throughout the compared putative polypeptides using *Ac*MNPV as a reference (Fig. 4). It is note worthy to mention that all *Spli*NPVs amino acid sequences (Acc# AAW49208, BAA00824, AAT10182 and AAW49207) were identical to *Polh-cr *putative polypeptide (Acc# AAR04375) except for the position 133 (I a.a. was replaced with V a.a.).

### Phylogenetic analyses of *Polh-cr*

**Figure 5:**
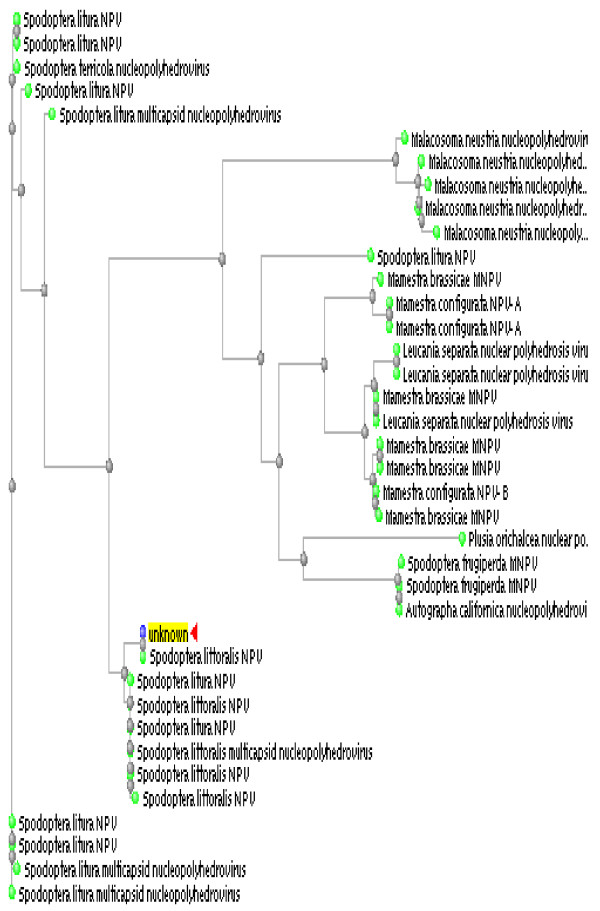
Phylogenetic analysis of *Polh-cr *nucleotide sequence compared to 38 published sequences. Unrooted tree was generated using ClustalW computer software. Full virus names are included in the tree.

**Figure 6:**
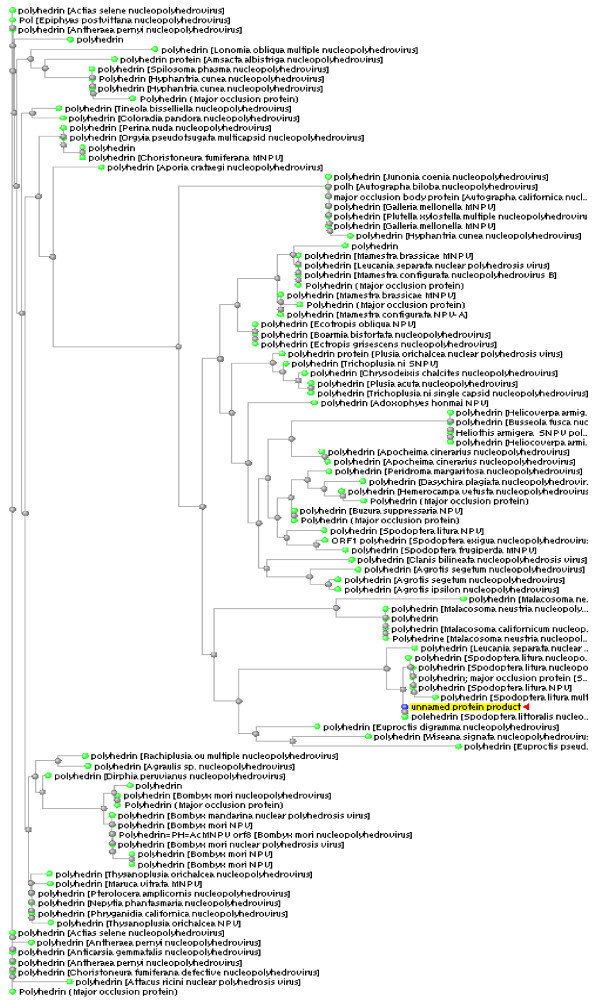
Phylogenetic analysis of *Polh-cr *deduced amino acid sequence compared to 102 published sequences. Unrooted tree was generated using ClustalW computer software. Full virus names are included in the tree.

Phylogenetic analyses have been performed on the *Polh-cr *nucleotide seuquence and its deduced polypeptide and results of these analyses are shown in Figs. (5 and 6). In case of *Polh-cr *nucleotide seuquence, a phylogenetic tree was generated from sequence data of 38 NPV isolates by neighbor-joining distance analysis with maximum sequence difference 0.75 (Fig. 5). The topology shows four distinct lineages including 5, 21, 8 and 4 NPV isolates, respectively. The maximum nucleotide sequence divergence was exhibited in lineage II. Meanwhile, the NPV isolates appear in the other three lineages as monophyletic sister clades (Fig. 5). In case of *Polh-cr *deduced amino acid seuquence, a phylogenetic tree was generated from sequence data of 102 NPV isolates by neighbor-joining distance analysis with maximum sequence difference 0.75 (Fig. 6). The topology shows three distinct lineages including 17, 60 and 25 NPV isolates, respectively. The maximum divergence of amino acid sequences was exhibited in lineage II. However, minimum divergence in case of the other two lineages was observed. Otherwise, polyhedrins from many NPV isolates appear in monophyletic sister clades (Fig. 6).

## Discussion

To date, genetically engineered baculoviruses introduce a promising research line to overcome the slow action of baculoviruses as biocontrol agents. On the other hand, searching for new natural baculovirus isolates with better insecticidal characteristics is still a developing subject of work (more safe and has not the risks of releasing genetically engineered product in nature). Although *Ac*MNPV is considered as the type species in the genus nucleopolyhedrovirus, 15 species and 471 tentative species of NPV have been isolated [27]. Abul-Nasr [28] isolated an Egyptian NPV from the cotton leaf worm *S. littoralis*. Cherry and Summers [29] isolated the two reference NPV types A and B. During 1986 – 1988, six natural isolates of *Spli*NPV were isolated from Giza, Menya, Kaha, Tokh, Kafr-Elsheikh and Gharbyia, Egypt (Khamiss, personal communication). Also, fifteen isolates of *Spli*NPV were isolated from the six above mentioned Egyptian localities in addition to Fayoum, Sakkara, Menofyia, Benisuef, Asyut, Sinnuris, Banha, Sharkyia and Elsaff-Giza, Egypt (Khamiss, personal communication).

Combining our data with that presented by Khamiss and by Seufi (personal communication), it can be concluded that 34 NPV isolates have been collected from Egypt between 1986 and 2000. This may reflect the suitability of the Egyptian environment for not only gathering and collecting new baculovirus isolates but also for using them as potential pesticides in integrated pest management (IPM) programs. Given that baculoviruses have been isolated from Upper as well as Lower Egypt, in which the cultivation was washed by chemical insecticides, it would be expected to find more and more isolates in virgin regions (where no insecticides were used).

The susceptibility of 2^nd ^instar larvae of *S. littoralis *to the two polyhedrovirus species reported in this study was comparable to that observed by Lacey and coworkers [22]. They reported that LC_50_s were 1.77 × 10^3 ^and 3.05 × 10^3 ^occlusion bodies (OB)/mm^2 ^for *Anagrapha falcifera *(*Af*MNPV) and *Autographa californica *(*Ac*MNPV) when applied to the neonate codling moth larvae. Our Egyptian isolate (*Spli*NPV) showed similar results to that obtained by Klein and Podoler against the Egyptian cotton leafworm [30]. Abot and coworkers [31] clarified that LC_50_s of an NPV isolate against two *A. gemmatalis *populations varied from 129 to 316 OBs/ml diet. Their results fall within the range obtained with our isolate. On the other hand, our results showed lower LC_50 _when compared with that reported by Pawar and Ramakrishnan [32] and Komolpith and Ramakrishnan [33] whereas LC_50 _was 4.677 × 10^6 ^PIB/ml for 4-day old *S. littoralis *larvae. Similarly, Ashok and Ramakrishnan [34] reported higher LC_50 _(7.1 × 10^6 ^PIB/ml) for 3-day old *S. litura *larvae. Also, Stiles and Himmerich [35] introduced higher LC_50 _of *Ac*MNPV against *H. zea *(3.46 × 10^4 ^– 6.38 × 10^5 ^PIB/ml). Finally, Abdel-Aziz (personal communication) presented higher LC_50_s (1.8 × 10^7 ^and 9.0 × 10^7 ^PIB/ml, respectively) for 2^nd ^larval instar of *S. littoralis*. The variability of LC_50_s is probably due to the method of surface treatment, homogeneously treated diet, feeding habit of the insect species [22] or due to difference in larval age [36,37]. It may also be due to difference in host susceptibility to NPV [4], number of virions per occlusion body, virulence of the virus strain and/or the difference in number of laboratory propagation cycles for the viral isolate. According to Van-Beek and Huges [38], the virulence of baculoviruses is best determined by the speed with which a given virus kills the insect pest.

LT values presented in Table (1) indicated that *Spli*NPV killed 2^nd ^instar larvae of *S. littoralis *one day faster than *Ac*MNPV. In comparing our results with that presented by other authors, many considerations have to be taken into account. Host range and LC_95_s of the viral isolates are the most important considerations. Although LC_95 _and LT_95 _that produced in this study are economically prohibitive, improving the insecticidal characteristics of such isolates (by formulation and synergistic additives) is a growing subject in many companies.

The full length of polyhedrin gene from lepidopteran NPVs ranged from 483 bp to 747 bp [39]. In case of polyhedrin gene from *Spodoptera *sp. NPVs, its full length ranges from 510 bp to 747 bp in comparison to that from *Autographa sp*. NPVs which ranges from 507 bp to 738 bp [39]. Therefore, it could be said that *Polh-cr *represented about 65% of the full length of polyhedrin gene. On comparing nucleotide sequence of *Polh-cr *to all available sequences in the GenBank, it created a significant homology with 100 NPV and 11 GV genes. It showed 99% identity with *S. littoralis *polyhedrin gene (Acc# D01017), 95% with *S. littura *polyhedrin gene (Acc# AY552474) and 93% with *S. littura *polyhedrin genes (Acc# AY549963, AF325155, AF037262 and AF068189). In addition, it was 90% similar to *Lymantria dispar *polyhedrin genes (Acc# AF499687, AF081810 and M23176), 88% to *Malacosoma neustria *polyhedrin gene (Acc# X55658) and 87% to *B. mori *polyhedrin genes (Acc# M10043 and X63614). Furthermore, it showed 86% homology with *S. litura *and *Amsacta albistriga *polyhedrin genes (Acc# X94437 and AF118850, respectively) and 85% with *S. exigua *and *Malacosoma neustria *polyhedrin gene (Acc# AF169823; AY127899 and AJ277555, respectively). These results ensured that *Polh-cr *is a highly conserved region within polyhedrin gene of about one sixth of the known NPV species. Consequently, it could be used in many molecular techniques concerned with baculoviruses.

Knowing that the full length of polyhedrin protein from lepidopteran NPVs ranges from 161 a.a. (e.g. *Hyphanteria cunea *NPV, Acc# AAW49190 and *Bombyx mori*, Acc# ABB16300) to 249 a.a. (e.g. *Spodoptera litura *NPVs, Acc# AAZ78353, NP_258269 and AAS90121). In case of *Spodoptera sp*. NPV, full length of polyhedrin protein ranges from 170 a.a. (Acc# AAW49204) to 249 a.a. (Acc# AAZ78353) in comparison to *Autographa *sp. NPV which ranges from 169 a.a. (Acc# AAW63393) to 246 a.a. (Acc# AAA46736). The deduced amino acid sequence of *Polh-cr *was compared to other polyhedrins. Alignment results revealed that *Polh-cr *was 100% and 99% identical to *S. littoralis *polyhedrins (Acc# AAC33752, AAR04375; P24646, JU0382 and BAA00824, respectively). It was also 99% similar to *S. litura *polyhedrins (Acc# NP258269, AAC09246, AAL01689 and AAS58468). In addition, it has 98% identity with polyhedrin of *S. littoralis *(Acc# AA590121). Furthermore, it showed 89% similarity with polyhedrins of *S. exigua *and *Ecotropis obligua *polyhedrins (Acc# AAF33532, JQ1868, NP037761, 001586; AAB53632, AAQ88174, P07388 and AAA46739).

Using *Ac*MNPV nucleotide sequence (Acc# M25054) and amino acid sequence (Acc# AAA46736) as references for comparison with our sequences, it was found that 57 different nucleotides and 12 gaps in nucleotide sequence resulted in 15 different amino acids in the putative polypeptide (I, N, V, V, E, D, S, T, A, T, S, D, K, Y and I a.a. from our putative polypeptide were replaced with V, S, I, I, D, E, V, N, S, H, E, N, R, E and V a.a. from *Ac*MNPV putative polypeptide, respectively). Surprisingly, *Polh-cr *putative polypeptide was identical to *Spli*NPV polypeptides (Acc# AAW49208, BAA00824, AAT10182 and AAW49207) with one amino acid replacement (I with V at the position 133). These results suggested the possible difference in codon usage among the compared isolates. It might also give a specific property to our putative polypeptide.

Phylogenetic analyses of the *Polh-cr *nucleotide seuquence and its deduced polypeptide revealed that *Polh-cr *is genetically related to a large number of published nucleotide sequences and to a larger number of published amino acid sequences. This finding made it is preferred to develop kits that use viral protein (polyhedrin) in detecting NPVs because it will be wider-used than DNA method.

## Conclusion

In this paper, we described the cloning and sequencing of a highly conserved region in polyhedrin gene and the insecticidal activity of an Egyptian NPV isolate. To our knowledge, this is the first report that determines the sequence of this conserved region in an Egyptian isolate. Further studies to develop kits for ELISA, Western and dot blotting, hybridization as well as potential biocontrol agent are switched on. The availability of *Polh-cr *products would be helpful in studies concerning ELISA, PCR and other related molecular techniques. In addition, it provides a candidate for effective, sensitive and reproducible diagnostic tools for screening insects or/and other arthropods, especially crustacean species, crabs and shrimps for baculovirus infections and may be important in controlling (preventing/enhancing) baculovirus infection. Also, it may be useful in monitoring the distribution of NPVs, the fate of genes and release of wild type as well as genetically engineered NPVs. Furthermore, it will facilitate risk assessment, ecological and viral epidemiological studies.

## Competing interests

The author(s) declare that they have no competing interests.
